# Molecular Characterization of Severe Acute Respiratory Syndrome Coronavirus 2 Isolates From Central Inner Sardinia

**DOI:** 10.3389/fmicb.2021.827799

**Published:** 2022-01-14

**Authors:** Paolo Malune, Giovanna Piras, Maria Monne, Maura Fiamma, Rosanna Asproni, Tatiana Fancello, Antonio Manai, Franco Carta, Giovanna Pira, Patrizia Fancello, Valentina Rosu, Antonella Uras, Caterina Mereu, Giuseppe Mameli, Iana Lo Maglio, Maria Cristina Garau, Angelo Domenico Palmas

**Affiliations:** ^1^UOC Ematologia, P.O. “San Francesco,” Azienda Tutela Salute, ASSL Nuoro, Nuoro, Italy; ^2^UOC Laboratorio Analisi Clinico-Chimiche e Microbiologia, P.O. “San Francesco,” Azienda Tutela Salute, ASSL Nuoro, Nuoro, Italy; ^3^UOC Cardiologia, P.O. “San Francesco,” Azienda Tutela Salute, ASSL Nuoro, Nuoro, Italy

**Keywords:** COVID-19, SARS-CoV-2, Sardinia—Italy, epidemiology, genome sequencing, phylogeny, pandemic (COVID-19), molecular characterization

## Abstract

**Background:**

The SARS-CoV-2 pandemic stimulated an outstanding global sequencing effort, which allowed to monitor viral circulation and evolution. Nuoro province (Sardinia, Italy), characterized by a relatively isolated geographical location and a low population density, was severely hit and displayed a high incidence of infection.

**Methods:**

Amplicon approach Next Generation Sequencing and subsequent variant calling in 92 respiratory samples from SARS-CoV-2 infected patients involved in infection clusters from March 2020 to May 2021.

**Results:**

Phylogenetic analysis displayed a coherent distribution of sequences in terms of lineage and temporal evolution of pandemic. Circulating lineage/clade characterization highlighted a growing diversity over time, with an increasingly growing number of mutations and variability of spike and nucleocapsid proteins, while viral RdRp appeared to be more conserved. A total of 384 different mutations were detected, of which 196 were missense and 147 synonymous ones. Mapping mutations along the viral genome showed an irregular distribution in key genes. *S* gene was the most mutated gene with missense and synonymous variants frequencies of 58.8 and 23.5%, respectively. Mutation rates were similar for the *S* and *N* genes with one mutation every ∼788 nucleotides and every ∼712 nucleotides, respectively. *Nsp12* gene appeared to be more conserved, with one mutation every ∼1,270 nucleotides. The frequency of variant Y144F in the spike protein deviated from global values with higher prevalence of this mutation in the island.

**Conclusion:**

The analysis of the 92 viral genome highlighted evolution over time and identified which mutations are more widespread than others. The high number of sequences also permits the identification of subclusters that are characterized by subtle differences, not only in terms of lineage, which may be used to reconstruct transmission clusters. The disclosure of viral genetic diversity and timely identification of new variants is a useful tool to guide public health intervention measures.

## Introduction

Severe acute respiratory syndrome coronavirus 2 (SARS-CoV-2) is a novel member of the genus Betacoronavirus (family Coronaviridae) and the causative agent of the coronavirus disease 2019 (COVID-19) pandemic (Zhou et al., 2020). To date (December 18th, 2021), WHO reports a total of 271,963,258 worldwide SARS-CoV-2 official cases and 5,331,019 COVID-19 deaths.^1^ Italy was one of the first European countries affected by the pandemic. The infection spread rapidly in Italy more than in other European countries (Maffeo et al., 2021). Italy responded well to the vaccination efforts, with 73.7% of the total population currently fully vaccinated.^2^ In Sardinia, the first case of a confirmed SARS-CoV-2 infection was reported on March 2nd, 2020, in a hospitalized patient at Cagliari hospital (Puci et al., 2020). According to a regional report from Istituto Superiore di Sanità of May 19th, 2021, a total of 56,158 cases of SARS-CoV-2 infection was reported in Sardinia, with a cumulative incidence of 3,513.77 cases every 100,000 people. The diffusion of the infection has not been homogeneous, and Sardinia -being a geographically isolated region- has the potential to show an isolated landscape in terms of viral diversity. Moreover, the situation is further complicated by the different travel limitations set in place throughout the pandemic, since the first few months -from March to May 2020- were characterized by strict travel limitations due to the national lockdown measures, while from May 2020 onward the afflux of people toward the island -a widely popular holiday destination- was no more limited.

Metagenomic massive parallel sequencing approach identified SARS-CoV-2 genome on January 7th and its entire 29.9 kbs RNA genome was readily shared on January 12th, giving invaluable input for research (Chen et al., 2020). The genome is structured as a long polyprotein-coding gene (*ORF1ab*) in the first two-thirds of the genome, which encodes 16 non-structural proteins (from nsp1 to nsp16). The remaining segment of the genome contains the *S*, *E*, *M*, and *N* genes, encoding the structural proteins, namely spike, envelope, membrane, and nucleocapsid proteins. While RNA viruses generally possess a higher mutation rate compared to DNA viruses, members of the Nidovirales order show a lower-than-expected mutation rate, due to the presence of a proof-reading activity in the RdRp (Llanes et al., 2020). Interestingly, the genome plasticity of SARS-CoV-2 was evidenced early in the pandemic with the detection of multiple sites under positive selection (Velazquez-Salinas et al., 2020). The acquisition of new mutations and natural selection results in mutant viral strains with increased infectivity and survival in the host environment. The substitution rate for SARS-CoV-2 is estimated to be in the order of 10^–3^/10^–4^ substitutions per site per year (Llanes et al., 2020). Therefore, understanding the genome changes of SARS-CoV-2 during the pandemic and their proper interpretations is critical for developing preventive, diagnostic, and therapeutic strategies against the virus as well for understanding its origin.

This study aims to characterize the circulating SARS-CoV-2 genomes in Nuoro province in terms of lineage and clade over time, highlighting differences from global frequencies from March 2020 to May 2021. The analysis also focuses on dissecting the complete viral genomes in terms of mutations, both in general and with special attention on the *S*, *N*, and *Nsp12*, which represent the best characterized SARS-CoV-2 genes.

## Materials and Methods

### Samples

RNA samples were collected as part of clinical diagnostics following official procedure (ISS Working Group Diagnostics and Microbiological Surveillance of COVID-19^3^). A total of 11.468 positive samples were collected in the context of the COVID-19 diagnostic workflow in “San Francesco” Hospital (Nuoro, Sardinia) from March 2020 until May 2021. A dataset of 92 SARS-CoV-2 complete genomes from subjects affected by COVID-19 was selected for this study among the SARS-CoV-2 genomic sequences registered in GISAID from our group and a few non-submitted ones, based on quality criteria of >400x coverage, >80% uniformity, and <1% N in the sequence.

All data used in this study was previously anonymized as required by the Italian Data Protection Code (Legislative Decree 196/2003) and the general authorizations issued by the Data Protection Authority. Ethics Committee approval was deemed unnecessary because, under Italian law, all sensitive data were deleted, and we collected only age, gender, and sampling date (Art. 6 and Art. 9 of Legislative Decree 211/2003).

### RNA Extraction and Reverse-Transcription PCR

Nasopharyngeal swabs were collected and placed in 3 ml of Universal Transport Medium (UTM, Copan Universal Transport Medium), transported at room temperature and tested for SARS-CoV-2 on the same day. Viral RNA was automatically extracted from 250 μl swabs medium using Seegene Nimbus system with the STARMag Universal Cartridge kit and subsequently tested using the Korea Ministry of Food and Drug Safety-approved Allplex 2019-nCoV assay (Arrow Diagnostics S.r.l., Genova, Italy), which detects the three target genes in a single-tube assay (*E* gene, *RdRp* gene, and *N* gene) as in the WHO-recommended protocols. Viral RNA aliquots from positive nasopharyngeal swabs were validated with the RealStar SARS-CoV-2 RT-PCR Kit 1.0, which detects the *S* gene. Ten nanograms of RNA (>1,000 virus genome copies) were used for whole viral genome sequencing.

### Severe Acute Respiratory Syndrome Coronavirus 2 Whole Genome Sequencing

Libraries were prepared with the Ion AmpliSeq Library Kit Plus according to the manufacturer’s instruction using Ion AmpliSeq SARS-CoV-2 RNA custom primers panel (ID: 05280253, Thermo Fisher Scientific). The RNA library preparation included reverse transcription using SuperScript VILO cDNA Synthesis Kit (Thermo Fisher Scientific) with subsequently 16–21 cycles of PCR amplification on the Ion Chef platform. Next-generation sequencing (NGS) reactions were run on Ion Torrent GeneStudio S5 sequencer.

Sequence alignments to the SARS-CoV-2 isolate Wuhan-Hu-1, complete genome (NCBI nucleotide collection, accession number: NC_045512.2) (Wu et al., 2020), was performed within the Torrent Server of Ion Torrent S5 sequencer using default settings. The aligned reads were utilized for both reference-guided assemblies. Assembly was performed using the Iterative Refinement Meta-Assembler (IRMA) v.0.6.1 (Shepard et al., 2016) that produced a consensus sequence for each sample using a >50% cutoff for calling single-nucleotide polymorphisms. IRMA utilizes multiple steps of alignment, variant calling, and consensus building by capitalizing on multiple allele frequency confidence intervals and read depth. Aligned reads were validated through the Integrative Genomics Viewer (IGV) v.2.9.4 (Thorvaldsdóttir et al., 2013).

### Genomic Characterization of Severe Acute Respiratory Syndrome Coronavirus 2 Sequences

SARS-CoV-2 sequences were analyzed in the Torrent Suite Software v5.12.1 by using the VariantCaller v5.12.0.4 and COVID19AnnotateSnpEff v.1.3.0.2 plugins, where mutations –with respect to SARS-CoV-2 reference isolate Wuhan-Hu-1 (NCBI nucleotide collection, accession number: NC_045512.2)- were detected and annotated. The sequences were then attributed to a PANGOLIN (Phylogenetic Assignment of Named Global Outbreak LINeages) lineage with PANGOLIN COVID-19 Lineage Assigner v.2.4.2 software (Rambaut et al., 2020) and to a NextStrain clade with the Nextclade v0.14.2 web tool (Hadfield et al., 2018). All queries were resolved with a PANGOLIN assignment conflict of 0. Next Strain classification helps to reference the origin of sequence patterns while the nomenclature of PANGOLIN provides a convenient scheme for detectable genomic introductions of SARS-CoV-2 into new regions. A picture of the percentage of the main circulating lineages in time and geographical location was obtained also by consulting the SARS-CoV-2 GISAID database. The global frequency and distribution of mutations in global GISAID depositions were obtained by consulting the CoVsurver mutations app in the GISAID website^4^.

Mutations detected by the VariantCaller plugin and subsequently annotated by the COVID19AnnotateSnpEff plugin for each sample were filtrated by allelic frequency (AF), retaining those with a value higher than 0.25.

### Phylogenetic Analysis

The 92 genomic dataset was aligned using MAFFT v7.479 webtool with default settings (Katoh et al., 2019). The alignment was visually inspected and manually curated using the Geneious Prime Software Java version 11.0.9 + 11 (Kearse et al., 2012). The Maximum Likelihood tree was generated on the corrected multiple alignment using the Mega X Software (Kumar et al., 2018) with the General Time Reversible model and the use all sites option. A total of 100 bootstrap replications were performed. The branches of the resulting tree were condensed using a cut-off of 70% bootstrap.

## Results

### Phylogenetic Analysis and Lineage Attribution

A total of 92 SARS-CoV-2 sequences (Table 1), collected from March 2020 to May 2021, were located in time according to sample collection and arbitrarily assigned to a I wave when collected in the period from March 2020 to June 2020 (*n* = 13), to a II wave from July 2020 to January 2021 (*n* = 42) and, finally, samples collected from February 2021 to May 2021 were assigned to a III wave (*n* = 37).

**TABLE 1:** Dataset of 92 Nuoro SARS-CoV-2 sequences.

Sample	Collection date	Coverage^a^	Uniformity^b^	GISAID	PANGO lineage
COV134	28/03/2020	435.4x	97.62%	EPI_ISL_637107	B.1
COV135	29/03/2020	4053x	99.70%	EPI_ISL_637108	B.1.1
COV130	31/03/2020	11957x	99.68%	EPI_ISL_614889	B.1.1
COV115	03/04/2020	10217x	99.48%	EPI_ISL_614398	B.1.1
COV129	03/04/2020	2768x	99.59%	EPI_ISL_458084	B.1.1
COV3	04/04/2020	6446x	99.58%	EPI_ISL_613560	B.1
COV5	05/04/2020	4638x	99.79%	EPI_ISL_613706	B.1.1
COV17	08/04/2020	641.4x	99.12%	EPI_ISL_613710	B.1
COV54	09/04/2020	6223x	96.71%	EPI_ISL_613955	B.1.1
COV21	10/04/2020	7744x	99.69%	EPI_ISL_613953	B.1.1
COV58	10/04/2020	834.7x	99.47%	-	B.1
COV171	19/04/2020	1889x	99.22%	-	B.1
COV92	28/04/2020	4197x	99.46%	EPI_ISL_614397	B.1.1
COV170	30/07/2020	16352x	98.94%	-	B.1.1.1
COV169	12/08/2020	12372x	99.87%	-	B.1.1.222
COV237	17/08/2020	6552x	99.66%	EPI_ISL_1191739	B.1
COV216	18/08/2020	6871x	99.37%	EPI_ISL_1191738	B.1
COV187	26/08/2020	3286x	99.33%	-	B.1.177.83
COV246	28/08/2020	9002x	99.38%	EPI_ISL_547965	B.1.177.83
COV297	04/09/2020	4656x	98.86%	EPI_ISL_1180250	B.1.367
COV299	04/09/2020	3456x	94.96%	EPI_ISL_1229145	B.1.177.83
COV193	07/09/2020	8519x	99.15%	EPI_ISL_1191736	B.1.177.83
COV194	07/09/2020	8761x	99.49%	-	B.1.177.83
COV207	08/09/2020	5285x	99.46%	EPI_ISL_1191737	B.1.177
COV284	14/09/2020	3876x	99.41%	EPI_ISL_1180251	B.1.177.83
COV305	17/09/2020	15334x	99.07%	EPI_ISL_1180252	B.1.177
COV307	19/09/2020	4071x	98.12%	EPI_ISL_1229144	B.1.177.83
COV401	06/10/2020	3743x	99.32%	EPI_ISL_1180253	B.1.177
COV503	07/10/2020	7404x	99.34%	EPI_ISL_1229146	B.1.177.75
COV502	16/10/2020	8741x	99.42%	EPI_ISL_965179	B.1.177.75
COV526	19/10/2020	14307x	99.33%	EPI_ISL_710503	B.1.177.75
COV731	23/10/2020	7165x	99.30%	EPI_ISL_1380060	B.1.177.83
COV733	23/10/2020	1064x	98.21%	EPI_ISL_1380061	B.1.177.51
COV734	23/10/2020	8368x	96.38%	EPI_ISL_1380062	B.1.177.50
COV735	23/10/2020	6662x	86.75%	EPI_ISL_1380063	B.1.177.83
COV757	23/10/2020	7819x	92.22%	EPI_ISL_1380064	B.1.177.83
COV758	26/10/2020	930,5x	90.05%	-	B.1.177.51
COV759	26/10/2020	6960x	80.93%	EPI_ISL_1380065	B.1.177.83
COV504	09/11/2020	4744x	99.33%	EPI_ISL_1229147	B.1.177.75
COV598	11/11/2020	4434x	99.34%	-	B.1.177.51
COV540	02/12/2020	3682x	98.53%	EPI_ISL_1229148	B.1.177.83
COV575	09/12/2020	526.9x	99.19%	EPI_ISL_1229149	B.1.177.50
COV2560	15/12/2020	7744x	98.98%	EPI_ISL_2098948	B.1.177.51
COV877	21/12/2020	5747x	99.28%	EPI_ISL_1191141	B.1.177.83
COV1241	31/12/2020	3057x	98.65%	EPI_ISL_1173202	B.1.1.7
COV850	13/01/2021	3129x	98.40%	EPI_ISL_1219710	B.1.177.83
COV841	15/01/2021	2782x	98.77%	EPI_ISL_1219707	B.1.177.83
COV843	18/01/2021	854,3x	98.37%	EPI_ISL_1219709	B.1.177.75
COV851	19/01/2021	426.1x	92.75%	-	C.16
COV849	20/01/2021	597.3x	97.50%	EPI_ISL_1219708	C.16
COV852	20/01/2021	465,3x	97.66%	-	B.1.177.83
COV880	21/01/2021	845.9x	96.42%	EPI_ISL_1191142	C.16
COV868	22/01/2021	8458x	99.01%	EPI_ISL_1191143	B.1.177.83
COV872	22/01/2021	1852x	98.43%	EPI_ISL_1191145	B.1.177
COV867	23/01/2021	5721x	99.31%	EPI_ISL_1191144	B.1.177.51
COV950	03/02/2021	470.8x	87.62%	-	B.1.177
COV953	03/02/2021	687.6x	96.82%	-	B.1.177
COV954	03/02/2021	1026x	97.32%	EPI_ISL_965114	B.1.177
COV955	03/02/2021	955.5x	95.94%	EPI_ISL_965028	B.1.177
COV996	03/02/2021	4014x	99.28%	EPI_ISL_965031	B.1.177.75
COV979	04/02/2021	4117x	99.55%	EPI_ISL_1841230	B.1.177.75
COV1089	06/02/2021	12371x	99.19%	EPI_ISL_1841231	B.1.177.75
COV1088	09/02/2021	9901x	99.26%	EPI_ISL_1841233	B.1.177.75
COV1077	12/02/2021	1457x	98.49%	EPI_ISL_1104650	B.1.177.75
COV1054	13/02/2021	3725x	88.57%	-	B.1.1.7
COV1060	13/02/2021	2482x	98.56%	EPI_ISL_1082253	B.1.177.51
COV1051	14/02/2021	11521x	99.41%	EPI_ISL_1034916	B.1.1.7
COV1091	14/02/2021	2490x	98.14%	-	B.1.1.7
COV1101	15/02/2021	2164x	98.35%	EPI_ISL_1063912	B.1.177.75
COV1202	24/02/2021	1703x	98.63%	EPI_ISL_1180141	B.1.1.7
COV1231	25/02/2021	10404x	99.82%	EPI_ISL_1180142	B.1.1.420
COV1282	26/02/2021	9514x	99.65%	EPI_ISL_1180143	B.1.1.7
COV1271	27/02/2021	1540x	98.74%	EPI_ISL_1180144	B.1.1.7
COV1682	17/03/2021	5892x	98.78%	EPI_ISL_1311860	B.1.1.7
COV1687	17/03/2021	5008x	98.19%	EPI_ISL_1311861	B.1.1.7
COV1691	17/03/2021	5583x	96.28%	EPI_ISL_1311862	B.1.1.7
COV1652	18/03/2021	900.4x	80.96%	EPI_ISL_1372546	B.1.1.7
COV1693	18/03/2021	5623x	98.50%	EPI_ISL_1372540	B.1.1.7
COV1889	26/03/2021	14505x	99.65%	EPI_ISL_1915606	B.1.1.7
COV2128	28/03/2021	13820x	99,53%	EPI_ISL_1557220	B.1.1.7
COV2141	29/03/2021	3506x	97.98%	EPI_ISL_1557221	B.1.1.7
COV2085	30/03/2021	13104x	99.67%	EPI_ISL_1557219	B.1.1.7
COV2137	30/03/2021	1903x	98.14%	EPI_ISL_1915607	B.1.1.7
COV2676	02/04/2021	30608x	98.06%	EPI_ISL_2091018	C.36
COV2480	13/04/2021	5384x	99.27%	EPI_ISL_2091457	B.1.1.7
COV2570	14/04/2021	11419x	99.31%	EPI_ISL_2280079	B.1.1.7
COV2634	23/04/2021	22893x	99.72%	EPI_ISL_2091016	B.1.351
COV2664	25/04/2021	6664x	99.14%	-	B.1.1.7
COV2678	29/04/2021	20232x	99.30%	-	B.1.1.7
COV2703	03/05/2021	20832x	99.17%	-	B.1.1.7
COV2697	07/05/2021	2785x	98.85%	-	B.1.1.7
COV2698	07/05/2021	1215x	93.81%	-	B.1.1.7

*The table presents each selected SARS-CoV-2 sample, date of collection, and associated sequencing quality values. For sequences that were deposited in the GISAID database EPICOVTM, the associated ID is given. The last column shows the attributed PANGO lineage.*

*^a^Coverage indicates the average sequencing depth, namely the average number of reads that cover each base in the target genome, averaged for the genome length.*

*^b^Uniformity represents the percentage of the genomic length that has been sequenced with a coverage that is at least 20% of the average value.*

**FIGURE 1:**
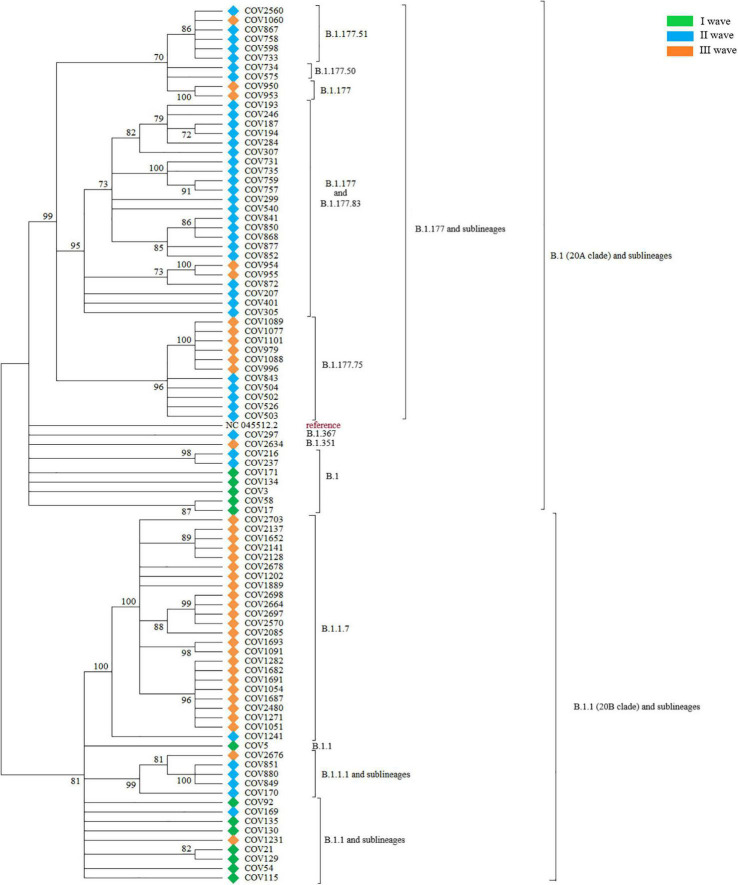
The figure shows the Maximum Likelihood phylogenetic tree obtained on the subset of 92 SARS-CoV-2 selected sequences. Major branches are annotated with the corresponding PANGO lineages and samples are labeled by wave.

As reported in Figure 1, the phylogenetic analysis identified a first major cluster supported by 81% bootstrap value, which further divides into a large subcluster (100% bootstrap) and two minor ones (99 and 82%, respectively). The other sequences outside of this main cluster also form another internal, big phylogenetic cluster, supported by 99% bootstrap, which subdivides into three clusters, supported by 70, 95, and 96% bootstrap, respectively. These main clusters all form other minor, internal subclusters. Within the main 81% bootstrap cluster, the large 100% bootstrap subcluster was populated by a subset of sequences belonging almost exclusively to the III wave. Sequences from the I wave were assigned to both the external group and the big cluster with 81% bootstrap, while sequences from the II wave were predominant in the 99% bootstrap cluster.

PANGOLIN lineage attribution was performed on the complete Nuoro dataset. The same sequences were then attributed to a NextStrain clade, to validate PANGOLIN results. The first major cluster supported by 81% bootstrap value was populated by all the sequences derived from the PANGOLIN lineage B.1.1 (corresponding to clade 20B). More in details, the majority of the sequences belonged to lineage B.1.1.7 and formed a phylogenetically supported internal cluster, plus some other minor branches that contained the sequences belonging to lineages B.1.1 and B.1.1.1, along with some other underrepresented sublineages, such as B.1.1.222 and B.1.1.420. The other sequences outside of this clade, instead, were attributed to lineages descending from lineage B.1 (clade 20A). More in details, this 20A group contains an internal, big phylogenetic cluster (supported by 99% bootstrap) that includes all sequences belonging to the PANGOLIN lineage B.1.177 and sublineages, plus a few minor branches containing sequences of lineage B.1, together with the two sequences attributed to lineages B.1.367 and B.1.351.

During the I wave, the circulating viral strains belonged only to the B.1 (38.5%, *n* = 5) and B.1.1 (61.5%, *n* = 8) lineages, according to the Italian epidemiology of pandemic. In Italy, in the same period, lineage B.1 was the prevalent one, with a frequency of 50%, followed by lineage B.1.1 with 30.1%. Other lineages were much less represented.

During the II wave, the situation was much more complex and heterogeneous. No B.1.1 sequence was found and only a small group of sequences belonged to the less mutated B.1 lineage and B.1.1 sublineages (11.9% in total, *n* = 5), while most of the circulating viral genomes were assigned to the B.1.177 lineage and derived sub-lineages (78.6%, *n* = 33). A few sequences (7.1%, *n* = 3) from the II wave in our dataset were attributed to the more divergent C.16 lineage. One sample (2.4%) of this wave was attributed to the B.1.1.7 lineage. In Italy, the situation was equally heterogeneous, with >50% of the sequences represented by lineages B.1.177 and sublineages. Lineage B.1.1.7 occupied 13.9% of the total sequences of this period, while the other lineages were about 5% of the total.

The III wave marked a decrease in the heterogeneity of circulating SARS-CoV-2 lineages in the Nuoro dataset, due to the widespread diffusion of the B.1.1.7 lineage, responsible for more than half of the total viral genomes for this period (62.1%, *n* = 23). A substantial, but markedly reduced, fraction of remaining sequences was identified as B.1.177 and related sub-lineages (29.7%, *n* = 11). Only one case of B.1.1 sub-lineage (B.1.1.420, 2.7%), apart from the B.1.1.7 lineage, was detected. Moreover, this wave marked the emergence, in our dataset, of more divergent lineages, such as B.1.351 (2.7%, *n* = 1) and C.36 (2.7%, *n* = 1). At this time, in Italy, lineage B.1.1.7 represented >75% of the total GISAID depositions. Other lineages remained equal or less than 5%.

When attributing the same sequences to a NextStrain clade, 8 clades were identified: 20A, 20B, 20C, 20D, 20E (EU1), 20H/501Y.V2 and 20I/501Y.V1. Almost all NextStrain clades in our dataset seem to respect some general correlations when compared to PANGOLIN lineages. Clade 20A included all B.1 sequences, while clade 20B included B.1.1, B.1.1.194, B.1.1.222, and B.1.1.420 lineages. B.1.367 was instead attributed to the 20C clade. Clade 20D included the B.1.1.1, C.16, and C.36 lineages. All the B.1.177 and sub-lineages were attributed to clade 20E (EU1). Lineages B.1.1.7 and B.1.351 were instead attributed to a dedicated clade, respectively, 20I/501Y.V1 and 20H/501Y.V2.

However, a notable exception to this general correlation was found, since COV1241 was assigned to the B.1.1.7 lineage by PANGOLIN COVID-19 Lineage Assigner v.2.4.2 and to the 20B clade by the Nextclade v0.14.2 algorithm. This conflicts with the observation that the B.1.1.7 lineage was otherwise always associated with 20I/501Y.V1 in our dataset, as expected considering the presence of the N501Y amino acid substitution -among all other typical ones- in the S protein.

### Mutational Analysis of 92 Severe Acute Respiratory Syndrome Coronavirus 2 Genomes

As reported in Table 2, a total of 384 different mutations were detected, compared to Wuhan-Hu-1 (NCBI NC_045512.2) reference sequence, of which 196 (51.0%) were missense mutations and 147 (38.3%) synonymous variants. Deletions and insertions (both in and out of frame) were 12 (3.1%) of the total, while upstream or downstream variants amounted to 25 (6.5%). Moreover, 4 stop codons (1.0%) were gained with a SNP in *ORF8*, *ORF3a* and *ORF7a*. When gene mutations were stratified by the wave of infection, missense mutations had a percentage of 50% (*n* = 12) in the I wave, 49.0% (*n* = 100) in the II wave, and 53.3% (*n* = 112) in the III wave, followed by synonymous variants with percentages of 37.5% (*n* = 9), 39.7% (*n* = 81), and 37.0% (*n* = 78), respectively. Deletion, insertions, and up-stream/downstream gene variants remained with a frequency below 10% in all three waves. The average number and standard deviation of mutations per sample in each wave were also calculated. During the I wave, 6.46 ± 1.20 mutations for each viral genome were observed, with an increasing trend during the following waves with average values of 19.43 ± 5.99 and 29.08 ± 5.95, respectively. The statistical significance of the increase in mutation number resulted in *p* < 0.05 (data not shown).

**TABLE 2:** Mutation types per wave.

	Nuoro dataset *N* = 92	I wave *N* = 13	II wave *N* = 42	III wave *N* = 37
Missense mutations	196	12	100	112
Synonymous variants	147	9	81	78
Deletions/Insertions	12	1	5	7
Up/Downstream variants	25	2	15	12
Stop gained	4	0	3	2

*Total mutations were identified, comparing the sequences with the reference NC_045512.2, annotated and clustered by type in the complete Nuoro dataset. Each mutation was considered only once independently by its frequency. Subsequently, mutations were divided by wave of collection of the sequence and clustered by type.*

Table 3 reported PANGOLIN lineages by timing of detection. In the I wave, the number of mutations was 6.80 ± 1.30 for B.1 and 6.25 ± 1.16 B.1.1. In the II wave, variants frequencies increased with 15.0 ± 2.83 mutations for B.1.177.50 and 19.82 ± 2.48 for B.1.177.83. Lineages B.1 and B.1.1 experienced an increase in mutations as well, with 9.50 ± 2.12 mutations for the B.1 samples. More late-emerging lineages, such as B.1.1.7 and C.16, were characterized by a higher number of mutations, 31 and 35 ± 1.73, respectively. In the III wave, B.1.1.7 was characterized by an average of 32.61 ± 1.99 mutations. The B.1.177 and sub-lineages of this wave presented a range value from 19.75 ± 1.71 to 21.33 ± 0.52 mutations, slightly more than what was detected in the previous time frame for the same lineages. The B.1.1.420 sample harbored 23 mutations and late-emerging lineages B.1.351 and C36 were characterized by 31 and 38 mutations when compared to the reference genome.

**TABLE 3:** Average mutations per lineage.

PANGO lineage	Average mutations	*S* mutations	*N* mutations	*Nsp12* mutations
**I wave**				
B.1	6.80 ± 1.30	1.0 ± 0	0	1.20 ± 0.45
B.1.1	6.25 ± 1.16	1.13 ± 0.35	1.0 ± 0	1.38 ± 0.52
**II wave**				
B.1	9.50 ± 2.12	1.0 ± 0	1.0 ± 0	2.0 ± 1.41
B.1.1.1	20	3	2	2
B.1.1.222	11	2	1	1
B.1.1.7	31	9	3	3
B.1.177	18.50 ± 1.73	4.25 ± 0.50	1.50 ± 0.58	1.0 ± 0
B.1.177.50	15.0 ± 2.83	2.50 ± 0.70	1.0 ± 0	1.0 ± 0
B.1.177.51	15.4 ± 2.07	2.0 ± 0	1.20 ± 0.45	1.0 ± 0
B.1.177.75	17.40 ± 2.30	3.60 ± 0.89	1.60 ± 0.89	1.0 ± 0
B.1.177.83	19.82 ± 2.48	4.24 ± 0.90	1.18 ± 0.39	1.59 ± 0.71
B.1.367	25	2	2	1
C.16	35 ± 1.73	5.33 ± 0.58	3.33 ± 0.58	4.0 ± 0
**III wave**				
B.1.1.420	23	5	3	2
B.1.1.7	32.61 ± 1.99	9.22 ± 0.60	3.09 ± 0.60	4.09 ± 0.42
B.1.177	19.75 ± 1.71	3.25 ± 1.50	2.0 ± 0	1.50 ± 0.58
B.1.177.51	21	3	2	1
B.1.177.75	21.33 ± 0.52	4.0 ± 0	1.0 ± 0	2.0 ± 0
B.1.351	31	10	1	1
C.36	38	9	2	3

*The table presents the average number and SD of mutations in the whole genome and in the S, N and Nsp12 genes for the sequences, clustered by lineage, from the Nuoro dataset.*

When the number of mutations per gene was counted only once independently by their frequency and clustered by type (data not shown), the *S* gene was the most mutated gene with missense and synonymous variants frequencies of 58.8 and 23.5%, respectively, while the same percentages for the *N* gene were 72.2 and 25.0%, respectively. The *Nsp12*, instead, counted 44.0% missense mutations and 56.0% synonymous variants. Mutation rates were similar for the *S* and *N* genes with one mutation every ∼788 nucleotides and every ∼712 nucleotides, respectively. *Nsp12* gene appeared to be more conserved, with one mutation every ∼1,270 nucleotides.

The most common amino acid substitution in the *S* gene, detected in every analyzed sequence of this dataset, was D614G (Table 4). In addition, other two widespread variants emerged during the II wave, A222V and P272L with frequencies of 52.4 and 50%, respectively. The III wave was characterized by the appearance of the subset of mutations that distinguish the B.1.1.7 lineage such as H69_V70del, Y144del, N501Y, A570D, P681H, T716I, S982A, and D1118H. Substitutions A222V, A262S, and P272L significantly deviated from the global distribution, demonstrating a higher prevalence in our dataset. A marked difference could also be observed for substitutions T696I, S884C, and Y144F. Mutations in the receptor binding domain RBD, from amino acid position 331 to 528, did not significantly deviate from global values, although substitutions identified in this region were globally rare (∼1% or less).

**TABLE 4:** Amino acid substitutions in spike, nucleocapsid and nsp12 proteins.

Spike	I wave (*n* = 13)	II wave (*n* = 42)	III wave (*n* = 37)	Global%
p.Ser12Phe	0	0	2.70%	0.35%
p.Leu18Phe	0	0	5.40%	6.09%
p.Ala67Val	0	0	2.70%	0.48%
p.His69_Val70del	0	2.38%	62.2%	45.51%–44.86%
p.His69_Ser71delinsPhe	0	0	2.70%	0.08%
p.Thr76Ile	7.69%	0	0	0.16%
p.Asp80Ala	0	0	2.70%	1.17%
p.Asp80Tyr	0	2.38%	0	0.23%
p.Tyr144Phe	0	11.90%	16.2%	0.08%
p.Tyr144del	0	2.38%	62.2%	44.13%
p.Trp152Arg	0	0	2.70%	0.69%
p.Asp215Gly	0	0	2.70%	1.16%
p.Ala222Val	0	52.4%	29.7%	8.93%
p.Arg237Lys	0	2.38%	0	0.01%
p.Leu242_Leu244del	0	0	2.70%	1.15%
p.Ala262Ser	0	31%	0	0.64%
p.Pro272Leu	0	50%	5.40%	0.47%
p.Arg346Ser	0	0	2.70%	0.06%
p.Lys417Asn	0	0	2.70%	1.27%
p.Asn440Lys	0	0	2.70%	0.14%
p.Leu452Arg	0	7.14%	2.70%	5.38%
p.Glu484Lys	0	0	2.70%	5.49%
p.Asn501Tyr	0	2.38%	62.2%	47.59%
p.Ala570Asp	0	2.38%	62.2%	44.90%
p.Asp574Tyr	0	0	2.70%	0.04%
p.Asp614Gly	100%	100%	100%	97.7%
p.Gln677His	0	0	2.70%	1.56%
p.Pro681His	0	2.38%	62.2%	47.13%
p.Ala684Val	0	2.38%	0	0.07%
p.Thr696Ile	0	9.52%	0	<0.01%
p.Ala701Val	0	0	2.70%	2.74%
p.Thr716Ile	0	2.38%	62.2%	44.89%
p.Thr732Ala	0	2.38%	0	1.23%
p.Pro812Ser	0	0	2.70%	0.15%
p.Ser884Cys	0	7.14%	0	<0.01%
p.Ala899Ser	0	0	2.70%	0.12%
p.Gly932Val	0	0	2.70%	<0.01%
p.Ser982Ala	0	2.38%	62.2%	44.09%
p.Thr1027Ile	0	2.38%	0	1.83%
p.Lys1073Asn	0	9.52%	0	0.12%
p.Arg1091Cys	0	2.38%	0	<0.01%
p.Asp1118His	0	2.38%	62.2%	44.12%
p.Ser1147Leu	0	2.38%	0	0.02%

**Nucleocapsid**	**I wave (*n* = 13)**	**II wave (*n* = 42)**	**III wave (*n* = 37)**	**Global%**

p.Asp3Leu	0	2.4%	27.0%	43.92%
p.Pro13Ser	0	0	2.7%	0.23%
p.Thr24Ile	0	0	2.7%	0.07%
p.Pro46Ser	0	0	5.4%	0.04%
p.Asp63Tyr	0	2.4%	0	0.05%
p.Asp98Asn	0	0	2.7%	<0.01%
p.His145Tyr	0	2.4%	0	0.24%
p.Thr148Ala	0	4.8%	0	0.01%
p.Ala156Ser	0	0	13.5%	0.24%
p.Ser180Ile	0	0	2.7%	0.04%
p.Ala182Ser	0	2.4%	0	0.13%
p.Ser186Tyr	0	2.4%	0	0.05%
p.Ser187Ala	0	7.1%	0	<0.01%
p.Ser197Thr	0	0	2.7%	0.05%
p.ArgGly203LysArg	61.5%	46.2%	67.6%	57.9%–54.37%
p.Thr205Ile	0	0	2.7%	5.49%
p.Gly212Val	0	0	2.7%	0.12%
p.Ala220Val	0	78.6%	29.7%	8.56%
p.Gln229His	0	2.4%	0	0.07%
p.Ser235Phe	0	2.4%	62.2%	44.40%
p.Pro326Leu	0	0	2.7%	0.04%
p.Asp377Tyr	0	2.4%	0	3.83%
p.Thr379Ile	0	0	5.4%	0.21%
p.Ala414Ser	0	0	2.7%	0.12%
p.Ser416Leu	0	2.4%	0	0.04%
p.Thr417Ser	0	0	2.7%	<0.01%

***Nsp12***	**I wave (*n* = 13)**	**II wave (*n* = 42)**	**III wave (*n* = 37)**	**Global%**

p.Thr4418Ile	0	0	16.2%	0.28%
p.Thr4477Ile	0	0	2.7%	0.12%
p.Asn4480Ser	15.4%	0	0	<0.01%
p.Thr4644Ile	0	0	5.4%	0.01%
p.Pro4656Thr	0	2.4%	0	0.04%
p.Pro4715Leu	100%	97.6%	100%	95.8%
p.Thr4794Ile	0	2.4%	0	0.01%
p.Ala4918Val	0	2.4%	0	0.07%
p.Ala4921Val	0	0	2.7%	0.27%
p.Leu5030Phe	0	2.4%	0	0.15%
p.Glu5136Asp	0	2.4%	0	0.11%

*The table presents the percentage frequency of all deletions/substitutions in the spike, nucleocapsid and nsp12 proteins, detected in the Nuoro subset. Deletions/substitutions were then stratified by wave. The last column shows the percentage frequency of each deletion/substitution in the complete set of total GISAID submissions.*

The most frequent mutation in the *N* gene (c.608_610delGGGinsAAC) was responsible for the R203_G204delinsKR double substitution, present in all three waves with percentages of 61.5, 46.2, and 67.6%, respectively. The II wave was also characterized by the appearance of the A220V substitution in 78.6% of cases. The III wave saw the introduction of the substitutions D3L and S235F. All the other substitutions were confined to a number of sequences inferior to 6. Table 4 also reports the global percentage frequencies, which showed that D3L, R203_G204delinsKR, and S235F were the most globally frequent ones, among the substitutions found in our dataset. Some significant deviations in our sequences, compared to the global values, are observed, such as A156S, S187A, and A220V. Almost all sequences in the three waves displayed the ubiquitous substitution P4712L, with only one exception, in the *Nsp12* gene. All the other substitutions were confined to 1 or 2 sequences only, with the only exception of T4418I, present in 6 cases (16.2%) of the III wave. Besides the ubiquitous P4715L, no other substitution found in our dataset displayed a percentage frequency higher than 1% in the global GISAID dataset.

Spike Y144F substitution was found in a subset of Nuoro samples that formed a supported subcluster among 20A clade members, and this internal subcluster was even further divided into two groups of sequences, attributed to the II (*n* = 5) and the III wave (*n* = 6), respectively. These sequences were all assigned to PANGOLIN lineage B.1.177.75. The analysis of the *S*, *N*, and *Nsp12* missense mutations highlighted how the Y144F sequences coming from the two waves also formed two sets in terms of mutations, where all the sequences from the III wave were characterized by the same missense mutations of the sequences from the II wave, with the addition of substitution T4418I in the RdRp and Y837H in the spike protein.

**FIGURE 2:**
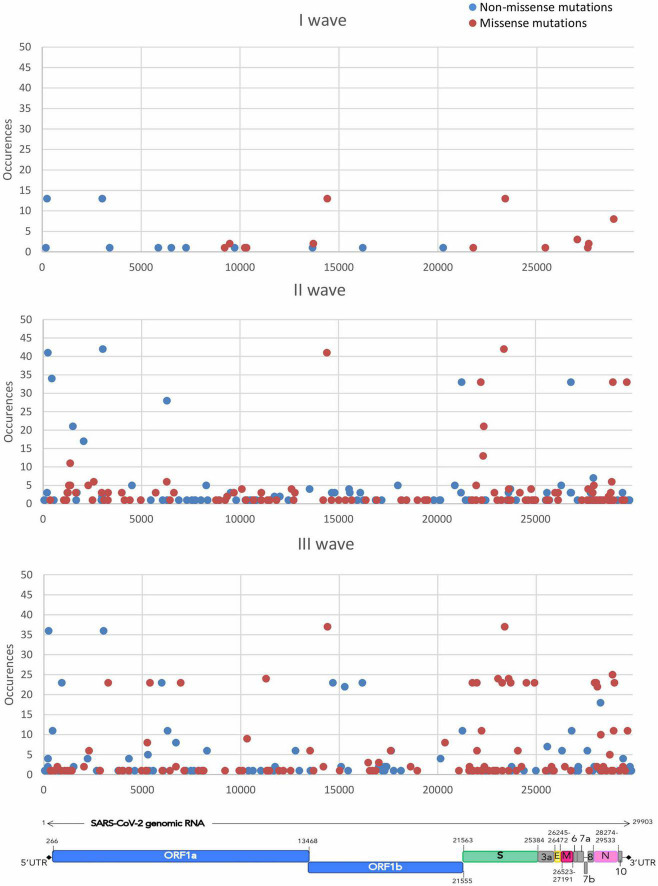
The figure shows the distribution of the accumulation of the different mutations throughout the SARS-CoV-2 genome over time. Missense mutations are shown in red and non-missense mutations, including synonymous, upstream and downstream variants, are shown in blue.

Mutation mapping across the SARS-CoV-2 genome (Figure 2) showed the presence of mutational prevalence hotspots, conserved throughout the three epidemic waves. In the first wave, missense mutations fell almost exclusively on *S* and *Nsp12* genes. The second wave experienced the circulation of common mutations in *Nsp*12, *S*, and *N* genes. During the third epidemic wave, newly introduced missense mutations occurred mostly in *S* and *N* genes together with other variations across the entire genome.

## Discussion

Worldwide effort to characterize SARS-CoV-2 and search for mutations while the virus continues to circulate can offer opportunities for a better understanding of viral evolution and transmission. This is why sequencing a large number of complete viral genomes has been crucial to monitor and analyze the circulating viral diversity in Nuoro province, which is characterized by the lowest population density in Italy and by a relatively isolated geographical location. The insular nature of Sardinia, along with the reduced number of residents, generates a condition that can produce very different epidemiological scenarios, compared to other areas.

Phylogenetic analysis revealed how the analyzed 92 SARS-CoV-2 sequences in the Nuoro dataset formed two main phylogenetically supported clusters, corresponding to lineages B.1 and by B.1.1 (and descendants), also associated with clade 20A and 20B, respectively. Besides this phylogenetic relation, SARS-CoV-2 genomes formed clusters connected with the timing of diffusion. Accordingly, sequences of the I wave -and hence associated with a low number of mutations- were found in both major groups, and sequences from this wave in the B.1 branch were found closer to reference NC_045512.2, as expected. Interestingly, the two sequences of the II wave and III wave attributed to the PANGOLIN lineages B.1.367 and B.1.351, despite harboring 25 and 31 nucleotide mutations as compared to the Wuhan-Hu-1/2020 reference sequence, respectively, were grouped together with the less mutated B.1 sequences of the I wave. The rest of the sequences of the II wave were indeed generally located in the 20A clade since this wave was mostly populated by B.1.177 and sublineages. Sequences from the III wave were instead generally located in the 20B branch, being mostly populated by B.1.1.7 members. In addition to these major clusters, several intra-lineage clusters were identified, likely attributable to transmission clusters.

When PANGOLIN lineages and NextStrain clades were assigned to the Nuoro sequences, it was possible to assess how the I wave, from March to June 2020, experienced the circulation of the two lineages B.1 and B.1.1 in a proportion of 38.5 and 61.5%, respectively. Consulting the depositions in the GISAID database for the same time window, among the 1,490 Italian submissions, 745 genomes (50%) were attributed to the B.1 lineage and 449 (30.1%) to the B.1.1 lineage. Our results differ from the Italian average data probably because general lockdown measures may have not allowed a proper homogenization of the viral diversity, favoring the creation and preservation of peculiar local landscapes. Moreover, the absence of the less frequently circulating lineages is to be expected in a smaller subset such as ours, for this wave. The sequences in the I wave were also distinguished by a relatively high degree of conservation, harboring a reduced set of mutations compared to the reference sequence, even though a remarkable local diversity was identified (Piras et al., 2021).

During the II wave, from July 2020 to January 2021, the average number of mutations experienced a 2–3-fold increase or more. This marked increase in mutation number within a few months, where a sequence collected at the end of April 2020 harbored only 7 mutations while sequences collected in July and August 2020 possessed up to 19–20 mutations, can be explained by the loosening of the lockdown measures that occurred during summer 2020 in Italy, which may be responsible for the introduction of a wider variability of viral strains. While Italy was one the European countries most severely hit, Sardinia was minimally affected during the I wave. However, being a famous holiday destination, the island experienced an important influx of tourists during summer 2020. The II wave was also marked by a decreased circulation of the more conserved B.1 and B.1.1 lineages and the emergence of the B.1.177 lineage and sub-lineages, which represented over 75% of the detections in the Nuoro province for the time window from July 2020 to January 2021. The diffusion of these lineages became significant during summer 2020 after they supposedly originated in Spain and diffused all over Europe after opening borders between countries (Hodcroft et al., 2021). Considerably, the only two B.1.1 sequences found in this time window were characterized by slightly more mutations compared to the B.1.1 sequences in the I wave, as well. The most common lineages in our dataset, namely B.1.177.75 and B.1.177.83 with a percentage of 11.9 and 40.5%, respectively, were mostly Italian lineages. Italy was responsible for the GISAID deposition of 62% of total B.1.177.83 sequences and 57% of total B.1.177.75 submissions. According to the GISAID database, B.1.177 sequences represented 32.6% of 4,068 total depositions from July 2020 to January 2021, while lineages B.1.177.75 and B.1.177.83 represented only 6.5 and 4.8%, respectively. Interestingly, lineage B.1.177.51, which was present with a percentage of 11.9% in our dataset, was only submitted 19 times on GISAID, during this timeframe. These marked differences can be explained by the presence of a bias, either because the circulation of the virus was different in Sardinia, due to its isolated geographical location, and/or because sample collection may have included multiple samples from the same clusters. Two other peculiar detections in this time windows were lineages C.16 and B.1.1.7, identified at the time, respectively, as variant under monitoring (VUM) and variant of concern (VOC) by the European Centre for Disease Prevention and Control, characterized by a particularly elevated number of mutations, compared to the other circulating lineages. A discrepancy emerged during the attribution of the NextStrain clade to sample COV1241 was explained when we identified two synonymous mutations present in all B.1.1.7 samples of the III wave and absent in COV1241. It emerged that these two synonymous mutations, namely c.14676C > T and c.15279C > T in *ORF1ab*, are included in the set of SNPs that are used by the Nextclade v0.14.2 algorithm to identify clade 20I. Moreover, sample metadata revealed that this was the first detection of lineage B.1.1.7 in Sardinia, dating back to December 2020. According to our data, this lineage did not significantly spread further for some weeks, reappearing in mid-February. Therefore, the genetic diversity of the first B.1.1.7 sample suggests multiple independent introductions of this lineage in Nuoro province.

The III wave, from February to May 2021, was distinguished by a reduction in the circulating viral heterogeneity in the Nuoro dataset, due to the prevalence of the B.1.1.7 lineage over the others, especially after March 2021, similarly to what shown by the worldwide epidemiology of pandemic. Considerably, lineage B.1.177.83, which was the prevalent one during the II wave, was not observed in this period, and no B.1 and less mutated sub-lineages (excluding, thus, lineage B.1.351) were found. It is interesting to notice how other more mutated lineages, such as B.1.351, C.36, and C.16 were found, along with the quite mutated B.1.1.7 itself, suggesting an adaptation of the virus to selective pressure. We observed how all B.1.177 and sub-lineages, as well as B.1.1.420, dated back to February and early March 2021, which marks the beginning of the identified III wave. No sequences attributed to these less recent lineages were found after mid-March 2021 in our dataset. It is important to also notice how most of the lineages detected in the III wave were then classified as VOC (B.1.1.7 and B.1.351) or VUM (C.36 + L452R) by the ECDC. Italian GISAID depositions are in line with these observations, with lineage B.1.1.7 representing > 75% of the detected genomes. The only important difference is the stronger persistence of B.1.177 and sublineages in the Nuoro dataset -especially lineage B.1.177. 75-, compared to Italian frequencies.

When mutation types, regardless of their frequency, were analyzed, a general conservation of the missense mutation percentages (51.0%) over synonymous ones (38.3%) in all three waves was observed, indicating again a trend of the virus toward a better fitness under selective pressure. Deletions, insertions, frameshift variants, and up/downstream variants were much rarer. This observation is also in line with the finding that the general number of mutations increased over time in the three waves. Interestingly, even though the number of different mutations increased over time, the ratio of missense over synonymous was generally preserved in all three waves.

Mapping the distribution and frequency of mutations throughout the genomic sequence displayed the presence of mutational hotspots. Some regions of the genome -from *Nsp4* to *Nsp10*, for instance- did not accumulate a significant number of mutations, while other genes -*Nsp12*, *S*, *N*- harbored mutations, both synonymous and missense, that were shared by a significant portion of the samples in the dataset. Subsequently the analysis focused on three essential protein-coding genes, namely *S*, *N*, and *Nsp12*. The analysis of gene-specific, frequency-independent mutation types highlighted how the ratio of missense over synonymous mutations is generally conserved between the *S* and *N* genes (2.5 and 2.9, respectively), while the *Nsp12* gene, instead, was characterized by a ratio of missense over synonymous of 0.79, indicating a higher degree of conservation of the sequence. It is evident how the *S* and *N* genes are less conserved, as confirmed by the frequency of accumulation of mutations per gene length, in the II and III waves, when the Nuoro SARS-CoV-2 circulating genomes started to differ from the reference genome. Thus, these two genes accumulated more missense mutations and conservative deletions/insertions compared to the *Nsp12* gene, which experienced no important increase in terms of accumulation of mutations over the three waves. Besides the spike protein, also the nucleocapsid protein is highly immunogenic and stimulates a potent humoral response (Smits et al., 2021), which could explain the retention of a higher number of substitutions in response to positive selection pressure for immune evasion. In contrast, the only currently used antiviral drug that targets the RdRp is still under investigation due to contrasting results regarding its efficacy, and in Italy its use is limited to hospital settings, as recommended by Italian Medicines Agency (AIFA), which may justify the low substitution rate and a higher degree of conservation compared to the other two proteins in response to poorer positive selection pressure.

The identification of the specific missense mutations for each gene highlighted how some substitutions are ubiquitous, such as spike D614G and RdRp P4715L (also known as P323L). Spike substitution D614G was associated with enhanced infectivity of SARS-CoV-2 (Hou et al., 2020), while no significant impact on neutralization with monoclonal antibodies and with sera from convalescent/vaccinated patients was detected (Weissman et al., 2021). RdRp P4715L was associated with a drastic change in protein structure and, possibly, function (Chand et al., 2020). It was also observed, along with spike D614G, to be more frequent in severely affected COVID-19 patients, compared to a cohort of mildly affected ones (Biswas and Mudi, 2020). Some globally rare (∼1% or less) spike substitutions in the RBD were identified, namely R346S, K417N, and N440K. In the *N* gene, a rather common missense mutation is responsible for the double substitution R203_G204delinsKR, which was frequently found worldwide and appears to have an important biological significance since it affects phosphorylation of nearby serines 202 and 206 in the nucleocapsid protein. These amino acids are included in a phosphorylation-rich site, which acts as a binding site for 14-3-3 proteins, which may work in altering nucleocapsid oligomerization during virion assembly (Tung and Limtung, 2020). Other substitutions were found in specific lineages, such as spike H69_V70del, Y144del, N501Y, or nucleocapsid S235F. All these substitutions were associated with the B.1.1.7 lineage, besides spike N501Y which also characterizes P.1 and B.1.351 lineages, as their corresponding clade nomenclature suggests. Spike N501Y was associated with a slight but significant reduction in neutralization (Wang et al., 2021). Double conservative deletion H69_V70del appears to be recurrent in multiple strains and it affects a defined antibody epitope, therefore possibly impacting immunity and/or antibody therapy (McCarthy et al., 2021). A mechanism of antibody evasion was identified *in vitro* when spike Y144 was deleted (McCallum et al., 2021). Other substitutions appeared confined to a small subset of sequences and have not been characterized so far.

Comparing the percentage frequencies of the substitutions in our dataset with the global values, some displayed a marked deviation. In particular, spike A222V, A262S, P272L, T696I, and S884C were significantly more frequent in our dataset. However, this is explained by the fact that all these substitutions were found in B.1.177 and sublineages samples, which were characterized by a prevalently European and Italian circulation and represented only a reduced fraction of global GISAID submissions. Another substitution whose frequency deviated from the global value was spike Y144F, which was again found only in sequences of the B.1.177.75 lineage. Globally, spike Y144F substitution was found quite rarely, with only 1,487 GISAID sequences on almost 2 million total depositions (<0.08%), but a significant proportion of this sequences (1.7%) came from Sardinia, indicating a higher prevalence of this mutation in the island. We speculate that this substitution, despite not being particularly radical, still removes a hydroxyl group in the VYY pocket, which may alter recognition and binding of specific antibodies. This pocket was identified as a potential T-cell epitope in a predictive bioinformatics analysis, which highlighted the disruption of the pocket structure when the Y144 residue was lost (Dawood et al., 2021).

In the *N* gene some deviations from the global frequencies were found as well, namely nucleocapsid A220V, which was again found only in B.1.177 and sublineages, or the A156S and S187A substitutions, which were associated with a few samples of the lineages B.1.1.7 and C.16, respectively, and only rarely found worldwide. In contrast, no other significantly diffused RdRp substitution was found in our dataset, except for P4717L. All other RdRp substitutions displayed a global percentage frequency < 1%.

In conclusion, we characterized the SARS-CoV-2 diversity in genomes circulating in the Nuoro province, finding a significantly increasing diversity over time, associated with the loosening of lockdown restrictions in summer 2020. The analysis of the viral genome in detail highlighted how *S* and *N* genes may be less conserved, how they evolved in time and identifying which mutations are more widespread than others. The high number of sequences also permits the identification of subclusters that are characterized by subtle differences, not only in terms of lineage, which may be used to reconstruct transmission clusters. The disclosure of viral genetic diversity and timely identification of new variants is a useful tool to guide public health intervention measures. Further studies are needed to characterize the landscape of the ongoing viral circulation in central inner Sardinia, which is expected to be dominated by B.1.617.2 and AY (Delta) lineages, and to track the spread of the worrisome B.1.1.529 (Omicron) lineage, which threatens to replace the currently circulating viral strains and -although no GISAID depositions in Sardinia are present to date- has been very recently detected in this region.

## Data Availability Statement

The data presented in the study are deposited in the GISAID repository (www.gisaid.org). The accession numbers are listed below: EPI_ISL_637107; EPI_ISL_637108; EPI_ISL_614889; EPI_ISL_614398; EPI_IS L_458084; EPI_ISL_613560; EPI_ISL_613706; EPI_ISL_613710; EPI_ISL_613955; EPI_ISL_613953; EPI_ISL_614397; EPI_ISL_ 1191739; EPI_ISL_1191738; EPI_ISL_547965; EPI_ISL_1180250; EPI_ISL_1229145; EPI_ISL_1191736; EPI_ISL_1191737; EPI_IS L_1180251; EPI_ISL_1180252; EPI_ISL_1229144; EPI_ISL_11 80253; EPI_ISL_1229146; EPI_ISL_965179; EPI_ISL_710503; EPI_ISL_1380060; EPI_ISL_1380061; EPI_ISL_1380062; EPI_I SL_1380063; EPI_ISL_1380064; EPI_ISL_1380065; EPI_ISL_12 29147; EPI_ISL_1229148; EPI_ISL_1229149; EPI_ISL_2098948; EPI_ISL_1191141; EPI_ISL_1173202; EPI_ISL_1219710; EPI_I SL_1219707; EPI_ISL_1219709; EPI_ISL_1219708; EPI_ISL_11 91142; EPI_ISL_1191143; EPI_ISL_1191145; EPI_ISL_1191144; EPI_ISL_965114; EPI_ISL_965028; EPI_ISL_965031; EPI_I SL_1841230; EPI_ISL_1841231; EPI_ISL_1841233; EPI_ISL_11 04650; EPI_ISL_1082253; EPI_ISL_1034916; EPI_ISL_1063912; EPI_ISL_1180141; EPI_ISL_1180142; EPI_ISL_1180143; EPI_I SL_1180144; EPI_ISL_1311860; EPI_ISL_1311861; EPI_ISL_13 11862; EPI_ISL_1372546; EPI_ISL_1372540; EPI_ISL_1915606; EPI_ISL_1557220; EPI_ISL_1557221; EPI_ISL_1557219; EPI_I SL_1915607; EPI_ISL_2091018; EPI_ISL_2091457; EPI_ISL_22 80079; and EPI_ISL_2091016.

## Author Contributions

GPs and MM conceived the study. MF and GM coordinated collection and processing the clinical samples. RA, FC, GPa, PF, and VR processed samples. PM and RA performed RNA isolation and RT-PCR. GPs, RA, PM, and AM performed NGS experiments. GPs, RA, PM, MM, and TF performed sequencing data analysis. PM analyzed NGS data with bioinformatics tools. AU and CM contributed to data analysis. PM and GPs prepared and wrote the manuscript. All authors contributed to generate the final version.

## Conflict of Interest

The authors declare that the research was conducted in the absence of any commercial or financial relationships that could be construed as a potential conflict of interest.

## Publisher’s Note

All claims expressed in this article are solely those of the authors and do not necessarily represent those of their affiliated organizations, or those of the publisher, the editors and the reviewers. Any product that may be evaluated in this article, or claim that may be made by its manufacturer, is not guaranteed or endorsed by the publisher.

## Footnotes

1
https://covid19.who.int/

2
https://vaccinetracker.ecdc.europa.eu/

3
https://www.iss.it/rapporti-covid-19/-/asset_publisher/btw1J82wtYzH/content/id/5329985

4
https://www.gisaid.org/epiflu-applications/covsurver-mutations-app
